# Relationship Between Wechsler Intelligence Scale for Children-IV Profiles and School Refusal in Children With Autism Spectrum Disorder

**DOI:** 10.7759/cureus.109855

**Published:** 2026-05-29

**Authors:** Mizue Kido, Tomomi Shinohara, Ichiro Ishikawa, Yu Nakamura

**Affiliations:** 1 Neuropsychiatry, Kagawa University Hospital, Miki, JPN; 2 Neuropsychiatry, Kagawa University School of Medicine, Miki, JPN

**Keywords:** autistic spectrum disorder, cognitive proficiency index, general ability index, school refusal, wechsler intelligence scale

## Abstract

Background

Autism spectrum disorder (ASD) is a neurodevelopmental disorder that has been increasing in prevalence in recent years and is associated with many comorbid disorders. Previous studies have reported that children with ASD are more likely to be truant than children without ASD. Issues related to learning, such as social problems and cognitive dysfunction, have also been identified.

Methodology

In this study, we investigated the relationship between the cognitive characteristics of children with ASD and school refusal. Children between 6 and 16 years old who visited our department from 2015 to 2022 and were diagnosed with ASD were included in the study. The subjects were divided into children who were attending school and those who were not attending school, and we examined their school attendance status along with the General Ability Index (GAI) and Cognitive Proficiency Index (CPI) using the Wechsler Intelligence Scale for Children-Fourth Edition. Of the 72 participants, 56 (77.8%) were included in the study.

Results

We included 25 participants (11 boys and 14 girls) in the school attendance group and 31 participants (18 boys and 13 girls) in the school refusal group. We examined the Full-Scale IQ (FSIQ) and composite index scores for both groups and found no significant differences in FSIQ, Verbal Comprehension Index (VCI), Perceptual Reasoning Index (PRI), Working Memory Index (WMI), Processing Speed Index (PSI), GAI, or CPI scores. However, the difference between the GAI and CPI scores was 1.16 ± 15.099 in the school attendance group and 9.48 ± 14.66 in the school refusal group. The difference between the GAI and CPI scores was greater in the school refusal group (*P* = 0.042).

Conclusions

The results of this study suggest the possibility that intra-individual differences in cognitive abilities may have an effect on school refusal of autistic children. Further research and studies are needed because there is a possibility that support methods in special-needs education will improve children's adaptation to school and learning efficiency.

## Introduction

School refusal

School refusal, as defined by Japan’s Ministry of Education, Culture, Sports, Science, and Technology, according to the definition adopted by the former Ministry of Education in the 1999 School Basic Survey, refers to non-attendance or absences exceeding 30 days per year owing to psychological, emotional, physical, or social factors, despite the child’s willingness to attend, excluding absences caused by illness or financial reasons. Similarly, Kearney describes school refusal as missing more than 25% of school time in the preceding two weeks, a noticeable struggle to attend during that period, or being absent for 10 days or for more than 15% of the time in the previous 15 weeks [1]. These definitions reflect the variations in how school refusal is understood across different countries.

In Japan, school refusal has emerged as a significant social issue, with a steady increase in cases over recent years. According to the Ministry of Education, the number of school refusals rose from 127,692 in 1998 to 299,048 in 2023. School refusal is a multifaceted problem, and various factors have been identified as its possible causes [2]. A study by Naylor et al. found that school refusers often have language disorders and learning disabilities, highlighting the connection between learning difficulties and school refusal [3]. This suggests that both individual factors and the learning environment are crucial in addressing this issue.

Autism spectrum disorder

According to the Diagnostic and Statistical Manual of Mental Disorders, Fifth Edition (DSM-5), autism spectrum disorder (ASD) is characterized by persistent deficits in social communication and interpersonal interactions across various contexts, as well as restricted, repetitive patterns of behavior, interests, or activities. A study by Kim et al. reported a recent ASD prevalence rate of 2.64%, a figure that has risen gradually, despite changes in diagnostic criteria [4]. In particular, children with high-functioning ASD are often overlooked during infant and toddler health checkups, and it is not uncommon for them to receive an ASD diagnosis only after they begin school, when they experience difficulties adjusting and stop attending.

Wechsler Intelligence Scale for Children-Fourth Edition

The Wechsler Intelligence Scale for Children-Fourth Edition (WISC-IV) is a well-established and effective tool for assessing cognitive abilities [5,6]. The Japanese version of the WISC-IV is a psychological assessment used to measure the intelligence of children aged 5 years 0 months (6 years 0 months in the U.S. version) to 16 years 11 months [7]. It comprises 15 subtests, divided into 10 core and 5 supplementary subtests [7]. The subtests are further categorized into four composite index scores - Verbal Comprehension Index (VCI), Perceptual Reasoning Index (PRI), Working Memory Index (WMI), and Processing Speed Index (PSI) - along with a Full-Scale IQ (FSIQ) that reflects general intelligence [7]. Furthermore, there are indices known as the General Ability Index (GAI) and the Cognitive Proficiency Index (CPI). The GAI is strongly correlated with the FSIQ index and reflects crystallized intelligence, verbal fluency, and nonverbal fluency abilities. By contrast, the CPI assesses the ability to efficiently process, including short-term memory and routine tasks. Although it does not represent the central aspect of intelligence, CPI is crucial for demonstrating both crystallized and fluid intelligence. Additionally, the fluency of information processing significantly influences intellectual activities, such as the acquisition of new knowledge. Prior studies have reported that children with ASD tend to have higher GAI scores compared with CPI scores [8]. Scorza et al. assessed the processing capacity of individuals with learning disabilities utilizing the FSIQ and GAI [9], while Kahalley et al. evaluated pediatric brain tumor patients by examining the differences between GAI and CPI [10]. There are previous studies on how GAI and CPI are used in research. 

Relationship between school refusal and ASD

John et al. and McClemont et al. reported that children with developmental disabilities, including ASD, mental disorders, and self-injurious behaviors, are at a higher risk of dropping out of school [11,12]. Additionally, sensory issues, such as gustatory hypersensitivity, may prevent some children from eating lunch at school. These findings suggest that developmental disabilities contribute to difficulties in learning and social adaptation at school.

According to Wenwen and Wenqiang, autism is associated with a cognitive profile of relative strengths in verbal and nonverbal reasoning and a weakness in processing speed, and autistic individuals might especially benefit from a cognitive assessment to identify and support their strengths and difficulties [13]. Caregivers commonly report in clinical settings that the individual demonstrates adequate adjustment when functioning at their own pace in both school and home environments; however, situations requiring participation in group activities or adaptation to others’ pace often lead to confusion and maladaptive behaviors. 

Therefore, this study formulated a hypothesis regarding individuals with high-functioning ASD, who are often overlooked during routine developmental screening. We evaluated the cognitive characteristics of children with ASD who refused to attend school.

In this study, we hypothesized that children with ASD who refused to attend school would exhibit a difference between their GAI scores, derived from the VCI and PRI, and CPI scores, calculated from the WMI and PSI. We investigated this retrospectively by analyzing their cognitive developmental characteristics to understand the factors behind school refusal better and identify early intervention strategies.

## Materials and methods

Methodology

Participants

**Figure 1:**
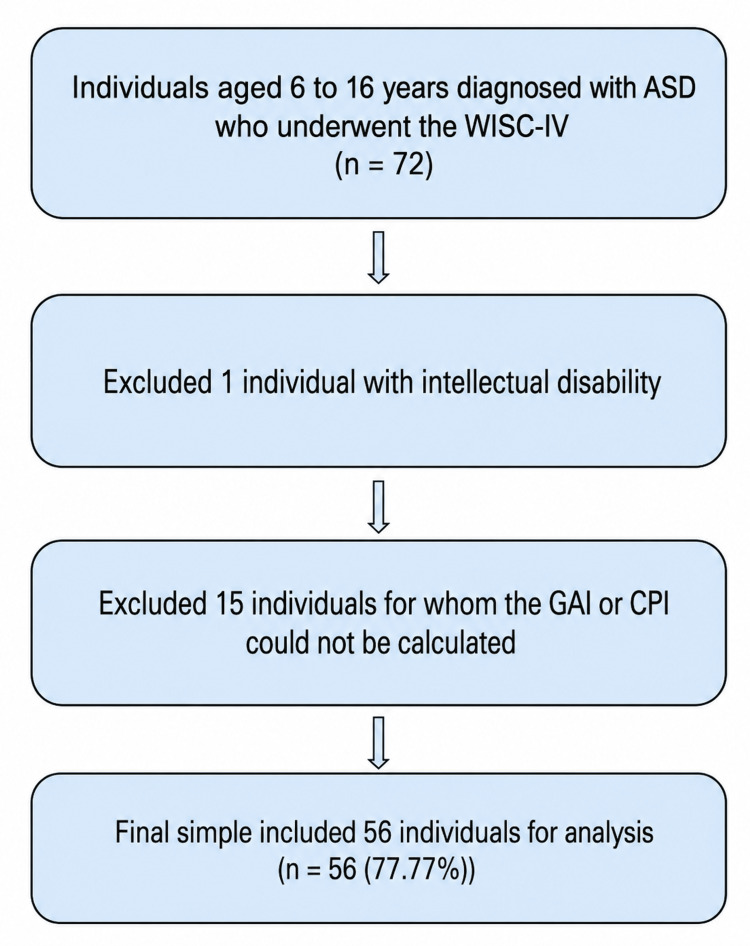
Process of selecting participants. Of the 72 participants, individuals with intellectual disability and those for whom GAI and CPI could not be calculated were excluded, resulting in a final sample of 56 participants. The figure was created by the authors using Google Docs. ASD, autism spectrum disorder; GAI, General Ability Index; CPI, Cognitive Proficiency Index; WISC-IV, Wechsler Intelligence Scale for Children-Fourth Edition

This study included children aged 6-16 years who first visited the Department of Neuropsychiatry, Kagawa University Hospital School of Medicine between January 2015 and July 2022 and were subsequently diagnosed with ASD by psychiatrists experienced in clinical management, based on the diagnostic criteria of the DSM-5. We categorized participants into the school attendance group and the school refusal group. The school refusal group was classified according to the above-mentioned definition provided by the Ministry of Education, Culture, Sports, Science, and Technology of Japan, “non-attendance or absence for >30 days per year because of inability, despite willingness, caused by psychological, emotional, physical, or social factors and backgrounds, excluding those caused by illness and economic reasons.” We reviewed participants’ medical records to verify school attendance and financial status. A total of 72 children underwent the WISC-IV assessment, and the results were analyzed retrospectively. Children with intellectual disabilities were excluded from the study. Participants with high-functioning ASD were enrolled in this study. Intellectual disability was excluded based on assessments of adaptive functioning and an IQ score below 70. We utilized the GAI and the CPI index scores for our evaluation; therefore, those for whom the GAI and CPI could not be calculated were excluded in accordance with the WISC-IV guidelines. For the administration and analysis of the WISC, we used *Using the cognitive proficiency index in psychoeducational assessment* [10]. As shown in Figure 1, 56 (77.77%) children were included in the analysis, comprising 29 boys and 27 girls, all of whom had calculable GAI and CPI scores. The mean age at the first visit was 10.63 ± 2.38 years. The demographic characteristics and WISC-Ⅳ profiles of all participants are detailed in Tables 1-2, respectively. 

Statistical analysis

Statistical analyses were conducted utilizing SPSS version 30.0.0.0(172) (IBM Corp., Armonk, NY). We performed a Shapiro-Wilk test to assess normality. For normally distributed data, we performed a Student’s t-test to compare the disparities between GAI and CPI scores for the school attendance and school refusal groups. Results are expressed as mean ± standard deviation, and a *P*-value of <0.05 was considered statistically significant. The GAI-CPI discrepancy was defined as the primary outcome measure, and statistical analyses were performed for the remaining variables and characteristic factors.

Ethical considerations

This study was conducted in accordance with the Declaration of Helsinki and received approval from the ethics committee of Kagawa University Hospital (approval number: 2022-134). Informed consent was not obtained as a result of the retrospective nature of the study; instead, we received approval from our hospital’s ethics committee and implemented an opt-out option. None of the participants refused to participate. We took precautions to protect personal data and managed the data to prevent the identification of participants. Furthermore, no companies that could pose a conflict of interest were involved in this study. Participants were informed of the study’s retrospective nature through our hospital’s homepage, and they were allowed to refuse participation in the present study. The authors declare that they have no conflicts of interest.

## Results

We included 25 participants (44.64%; 11 boys and 14 girls) in the school attendance group and 31 participants (55.35%; 18 boys and 13 girls) in the school refusal group. The mean ages at the first visit were 10.72 ± 2.47 years in the school attendance group and 10.54 ± 2.33 years in the school refusal group (Tables 1-2).

**Table 1:** Background of all participants. The total number of participants was 56, and there was no difference in the rates of school attendance and school refusal between boys and girls. (Sex distribution was compared using Fisher’s exact test, and age was compared using the chi-square test.)

Patients’ characteristics	All patients	School attendance group	School refusal group	*P*-value
Number of participants (male), *n* (%)	56 (29)	25 (11)	31 (18)	0.21
Age at first visit (years)	10.63 ± 2.38	10.72 ± 2.47	10.54 ± 2.33	0.71

**Table 2:** WISC-Ⅳ composite score profiles of all participants. For both the FSIQ and the index scores, the mean FSIQ and composite index scores were within the average range. FSIQ, Full-Scale Intelligence Quotient; VCI, Verbal Comprehension Index; PRI, Perceptual Reasoning Index; WMI, Working Memory Index; PSI, Processing Speed Index; GAI, General Ability Index; CPI, Cognitive Proficiency Index; WISC-Ⅳ, Wechsler Intelligence Scale for Children-Fourth Edition

Index	Minimum value	Maximum value	Mean value	Standard deviation
FSIQ	71	134	98.41	13.37
VCI	58	133	99.80	14.56
PRI	66	141	101.46	16.23
WMI	71	133	97.00	14.91
PSI	67	127	94.48	12.31
GAI	62	143	100.75	15.71
CPI	70	121	95.80	12.24

**Figure 2:**
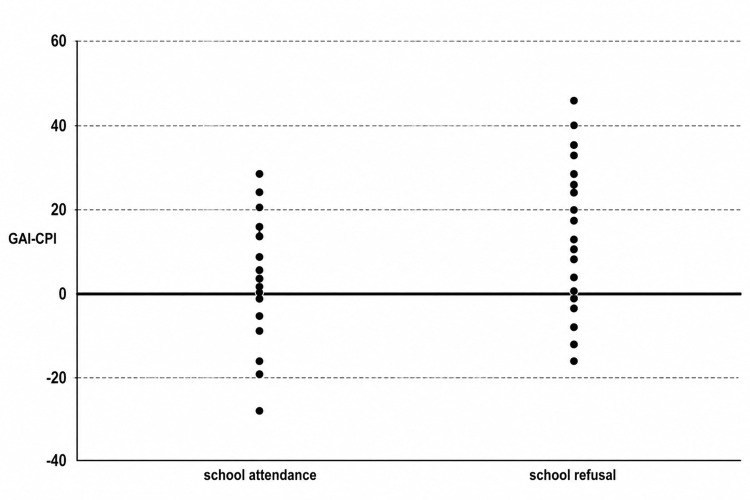
Independent dot plot of GAI-CPI for the school attendance and school refusal groups. GAI-CPI, General Ability Index-Cognitive Proficiency Index

We examined the FSIQ and composite index scores for both groups and found no significant differences in FSIQ, VCI, PRI, WMI, PSI, GAI, or CPI scores (Table 3). However, the difference between the GAI and CPI scores was 1.16 ± 15.099 in the school attendance group and 9.48 ± 14.66 in the school refusal group (Table 4), as analyzed using Student’s t-test. Furthermore, the findings indicated that the GAI score was higher than the CPI score in the school refusal group (Figure 2). Notably, the difference between the GAI and CPI scores was significantly greater in the school refusal group (*P* = 0.042) (Table 4).

**Table 3:** Comparison of WISC-IV composite scores between the school attendance and school refusal groups. This table presents the full-scale intelligence quotient (FSIQ) and WISC-IV composite index scores for the school attendance and school refusal groups. Statistical analyses were performed using Student’s t-test. A *P*-value < 0.05 was considered statistically significant. WISC-IV, Wechsler Intelligence Scale for Children-Fourth Edition

Index classification		*n* (%)	Mean value	Standard deviation	Standard error of the mean	*t*-value	*P*-value	Difference in means	Standard error of difference	95% confidence interval of the difference	
										Lower limit	Upper limit
FSIQ	Attendance	25 (11)	98	12.29	2.45	-0.20	0.83	-0.74	3.62	-8.015	6.53
	Refusal	31 (18)	98.74	14.38	2.58						
VCI	Attendance	25 (11)	98.76	14.10	2.82	-0.47	0.63	-1.88	3.94	-9.78	6.018
	Refusal	31 (18)	100.65	15.096	2.71						
PRI	Attendance	25 (11)	99.32	14.32	2.86	-0.88	0.38	-3.87	4.37	-12.64	4.89
	Refusal	31	103.19	17.66	3.17						
WMI	Attendance	25 (11)	97.56	14.99	2.99	0.25	0.80	1.012	4.044	-7.096	9.11
	Refusal	31	96.55	15.08	2.709						
PSI	Attendance	25 (11)	96.64	12.17	2.43	1.18	0.24	3.89	3.29	-2.71	10.50
	Refusal	31 (18)	92.74	12.33	2.21						
GAI	Attendance	25 (11)	99.08	14.26	2.85	-0.71	0.48	-3.017	4.24	-11.52	5.49
	Refusal	31 (18)	102.1	16.90	3.03						
CPI	Attendance	25 (11)	97.92	12.56	2.51	1.16	0.24	3.82	3.28	-2.75	10.40
	Refusal	31 (18)	94.1	11.90	2.13						

**Table 4:** Comparison of GAI-CPI indices between the school attendance and school refusal groups. There was a significant difference in GAI-CPI between the groups. Statistical analyses were performed using Student’s t-test. A *P*-value < 0.05 was considered statistically significant. GAI-CPI, General Ability Index-Cognitive Proficiency Index

GAI-CPI	*n* (%)	Mean value	Standard deviation	Standard error of the mean	*t* value	*P*-value	Difference in means	Standard error of difference	95% confidence interval of the difference	
									Lower limit	Upper limit
Attendance	25 (11)	1.16	15.099	3.02	-2.084	0.042	-8.32	3.99	-16.33	-0.31
Refusal	31	9.48	14.66	2.63						

## Discussion

In this study, we assessed the cognitive functional characteristics of children with ASD using the GAI and CPI, along with the difference between these indices. Although no significant differences were found in the scores for FSIQ, VCI, PRI, WMI, or PSI between the school attendance and school refusal groups, the difference between the GAI and CPI scores was greater in the school refusal group.

IQ scores are often used to predict academic performance; however, discrepancies between the GAI and CPI indicate the low predictive validity of the FSIQ [14]. Stephenson et al. reported that the GAI score generally tends to be higher compared with the CPI score in children with ASD [15]. Prior meta-analyses have exhibited that individuals with ASD demonstrate significantly poorer working memory compared with their neurotypical peers, regardless of age [16,17]. The CPI serves as an effective measure of information processing derived from working memory and processing speed tasks [13]. In this study, while there was no difference in GAI or CPI scores between the two groups, a discrepancy between GAI and CPI was observed, particularly among children who did not attend school. This raises the question: What does a difference between GAI and CPI indicate? In neurodevelopmental disorders, variability in cognitive characteristics within individuals is considered a contributing factor to difficulties in daily living. Prior research has suggested the possibility that a low CPI relates to poor social adaptation skills. Kim et al. suggested that PSI may predict timed academic performance [18]. They reported that PSI affects timed academic performance independently of FSIQ, suggesting that cognitive abilities may have an impact on academic performance that cannot be fully predicted by FSIQ alone. The findings of this study imply that the difference between GAI and CPI may also signify challenges in daily living and social participation. Moreover, the lack of significant difference between GAI and CPI scores in the school attendance group suggests that some children with ASD may not exhibit substantial discrepancies between higher brain functions such as reasoning, knowledge, and conceptualization and cognitive functions that support the entire intellectual activity, potentially indicating a lower likelihood of developing social maladaptive behaviors.

Limitations

The first limitation of this study concerns the characteristics of the study population. The findings may have been influenced by the fact that many participants did not have intellectual impairments. Therefore, further research is warranted to clarify the cognitive profiles of children with ASD who attend support classes while receiving academic assistance. Additionally, Rosen et al. indicated that symptoms associated with adjustment to school life differ between high-functioning ASD and those with intellectual disabilities [19]. Regarding the intellectual level of children with ASD, Rosa et al. reported that low IQ is associated with internalizing disorders [20], whereas Greenlee et al. found that high IQ is linked to depression [21]. Furthermore, the results of this study are limited to participants who visited our hospital and underwent testing, which may not fully represent the broader ASD population, and the sample size of 56 participants may further limit the generalizability of the findings. The second limitation of this study is about school refusal as a phenomenon. School refusal is influenced by various factors, including academic pressures and external circumstances such as parental conditions [22]. The third is about ASD. Cognitive abilities in children with ASD are influenced by various factors. Indeed, several factors that should be taken into consideration, such as ASD subtype, rehabilitation history, age, sex and school-based support system. In addition, it will be necessary to clarify the impact of other comorbid symptoms of ASD, such as sensory hypersensitivity and anxiety or depression resulting from secondary conditions. Since the educational environment may also influence the WISC-IV, it is necessary to collect such information and conduct a review. To obtain more robust and generalized results, future studies should consider the contextual factors associated with ASD. Qiao and Wang reported an association between cognitive abilities in children with ASD and factors such as sex and age that gender differences were evident in PSI, with girls outperforming boys, while age differences were minimal [13]. This discrepancy is influenced by factors such as ASD subtype, rehabilitation history, and parenting circumstances.

Therefore, although the this study suggests an association between school refusal and the discrepancy between GAI and CPI, several issues remain to be addressed before these findings can be applied to educational support and interventions for school refusal in children with ASD. It is hoped that future research will further clarify effective educational approaches tailored to the cognitive characteristics of individuals with ASD; however, further investigation is needed to explore this relationship.

## Conclusions

In this study, we retrospectively examined and discussed the results of the WISC-IV assessments conducted for children with ASD. Additionally, we explored differences in WISC profiles between the school attendance and school refusal groups, focusing on GAI and CPI. The WISC-IV is a widely used general intelligence test, and this study demonstrates its potential to address school refusal through this versatile psychological tool. Given that school life requires various skills, including social interactions with peers and teachers, as well as academic abilities, the findings suggest the possibility that leveraging GAI and CPI may facilitate improved school adaptation for students in the future.
