# Correction: Relationship Between Wechsler Intelligence Scale for Children-IV Profiles and School Refusal in Children With Autism Spectrum Disorder

**DOI:** 10.7759/cureus.c470

**Published:** 2026-08-11

**Authors:** Mizue Kido, Tomomi Shinohara, Ichiro Ishikawa, Yu Nakamura

**Affiliations:** 1 Neuropsychiatry, Kagawa University Hospital, Miki, JPN; 2 Neuropsychiatry, Kagawa University School of Medicine, Miki, JPN

The Table 4 legend has been corrected to state: “There was a significant difference in GAI-CPI between the groups.” Previously this was misstated as no significant difference.

